# Supplementary material for: Are carbon emissions trading and green financial instruments synergistic? -Comprehensive quantitative research based on content analysis
Source: PLoS One. 2024 Mar 7;19(3):e0298601. doi: 10.1371/journal.pone.0298601 (PMC10919601; doi:10.1371/journal.pone.0298601)
Supplement: S1 Data — (ZIP) [file pone.0298601.s001.zip › Data/地级市Z_F工作报告.zip!/║■▒▒/╛ú╓▌╩╨/└┤╫╘╣1⁄2╓┌║┼í╛┬φ┐╦╩2╛▌═°í┐_2017.docx]

各位代表：

现在，我代表市人民政府向大会作工作报告，请予审议，并请各位政协委员和列席人员提出意见。

一、2016年和过去六年工作回顾

2016年，是“十三五”的开局之年，也是壮腰工程“五年大跨越”的见效之年。面对经济下行压力和特大洪涝灾害双重考验，在省委、省政府和市委的领导下，在市人大和市政协的监督支持下，我们团结全市人民，深入贯彻落实习近平总书记系列重要讲话精神，主动适应经济发展新常态，负重奋进、砥砺前行，经济社会发展呈现“稳中有进”的良好态势。初步核算，实现地区生产总值1726.8亿元、增长7.3%。完成固定资产投资2001亿元、增长18%；规模以上工业增加值增长6.8%；社会消费品零售总额1056亿元、增长11.6%；地方公共财政预算收入115.45亿元、增长11.6%；城乡居民人均可支配收入分别达到27666元、14707元，增长9%和7.1%。

过去一年，我们主要做了以下工作。

一是主动应对下行压力，保持了经济增长。

实体经济发展稳健。开展“万名干部进万企”活动，在全省率先出台供给侧结构性改革“三去一降一补”行动方案，设立长江经济带壮腰工程等3只产业基金，继续实施支柱产业倍增、骨干企业壮大、中小企业成长工程，全市规模以上工业企业达到1240家、净增99家。实施“五证合一、一照一码”，新增市场主体5万户。

项目落地建设提速。亿元以上工业项目完成投资650亿元，葛洲坝水泥产能置换、长利玻璃、能特科技、沃特玛新能源产业园等项目竣工投产。华电江陵发电厂、荣成再生科技、香港国际家居产业园等项目进展顺利。达鑫电子、爱斯达智能服装等项目落地开工。完成交通投资210亿元，江北高速、武汉城市圈环线洪湖段、荆松一级公路、荆当旅游公路基本建成，石首长江大桥、洪湖嘉鱼长江大桥、沙公高速、318国道荆州城区段改线等项目建设加快。PPP入库项目57项、总投资778亿元。

消费业态拓展加快。电商零售额130亿元、增长62%，电商企业加速发展，覆盖行政村超过500个，岑河婴童装上线“中国质造”。扩大公积金使用范围，鼓励引导农民进城购房，全市实现商品房销售4.6万套，销售总额220亿元、增长53%。成功举办第三届中国汽车（房车）露营大会，松滋洈水获批全国首个五星级汽车自驾运动营地。

二是坚持改革开放并举，增强了发展后劲。

各项改革稳步推进。建立“四单一网”。清理规范“红顶中介”215家。推进荆州开发区“一区多园”管理体制改革，“一枚印章管审批、一支队伍管执法”改革取得突破。深化投融资体制改革和国企改革，南湖机械与中国航天科工实现重组联合。“营改增”全面完成，国地税征管合作机制改革领跑全省。党政群机关公车改革基本完成。机关事业单位养老保险制度改革顺利推进。不动产登记发证实现全覆盖。

对外开放力度加大。新引进亿元以上项目363个，其中工业项目337个，实际到位资金1578亿元。新增外资企业10家，实际利用外资1.44亿美元、增长8%。外贸出口总额9.5亿美元。荆州开发区纳入长江经济带国家级转型升级示范区。对外开放口岸纳入国家“十三五”规划，综合保税区启动申报程序，荆州港获批国家指定粮食进口口岸。古城保护与修复世行贷款项目顺利启动。

三是着力推动结构调整，加快了转型步伐。

农业固本提质。全力抗灾复产，粮食提质稳产的基础进一步巩固。淡水产品、油菜籽产量保持全国市州第一。农产品加工业总产值1550亿元，规模以上企业净增30家、达到542家。新型农业经营主体4.2万个，总量全省第一。“荆楚味道”走俏湖北、迈向全国。有效使用的“三品一标”产品达到429个。省水产产业技术研究院落户荆州。华中农高区争创国家级农高区取得进展。洪湖市获评“中国名蟹第一市”。松滋市荣获国家农产品质量安全示范市。

工业提档升级。高新技术企业达到140家，实现增加值165亿元，占GDP比重9.8%。美的智能冰箱、五方光电二期、凯乐数据链通讯等高新技术项目投产见效。装备制造、医药化工、电子信息产值分别达到529亿元、225亿元、189亿元。税收过千万的工业企业56家，其中过亿元的企业8家。白云边酒业入库税金7.8亿元，连续七年获评“鄂企百强”。

服务业增长较快。商贸服务业“个转企、小进限”力度加大，限额以上商贸企业净增35家、达到967家。关公义园建成开放，投资120亿元的华夏历史文化科技园、投资28亿元的海洋世界等项目开工建设，楚王宫、“楚国八百年”城市公园等项目有序推进，全市在建重大旅游项目22个。荆州楚王车马阵景区、松滋洈水旅游区获批国家4A景区。旅游综合收入200亿元、增长21%。县域金融工程全面发力， “新三板”挂牌企业新增4家，“四板”挂牌企业新增192家。公安县农村“两权”抵押贷款在全国首批试点。

四是统筹城乡一体化发展，提升了城市形象。

城市创建力度加大。荆北、沙北片区竞相发展，荆州开发区、纪南文旅区、华中农高区三大功能区建设加快。中心城区新修、改建主次干道和支街小巷78千米，新建给排水管网170千米，新开工棚改项目36个。荆州市沙市实验小学新校区建成，荆州中学、沙市中学、荆州体校主体工程竣工，市图书馆新馆、市公共检验检测平台一期基本建成，荆州职业技术学院、鄂中南区域医疗中心、市一医门诊综合大楼等项目开工。荆江园、万寿公园提档升级，临江仙主体公园配套、荆江大堤堤顶黑化、环城北路建设全面完成。国家园林城市通过复检，国家卫生城市创建取得阶段性成果，成功创建省级森林城市。

新型城镇化步伐加快。市县“多规合一”试点有序推进，城镇发展体系规划基本完成。松滋国家新型城镇化综合试点顺利推进，卸甲坪乡成为全域旅游、美丽乡村建设的样板。监利新沟等11个“四化同步”试点镇建设加快。通信基础设施投入9.2亿元，电网建设完成投资19.9亿元。农村水、电、路、气等基础设施建设加快。

五是落实绿色发展理念，加强了环境保护。

实施“三禁两治”“三污同治”，严打“三非”，年度节能减排任务全面完成。重拳整治环保突出问题，清理整顿违法违规项目806个。积极推进“河长制”“湖长制”。启动自然资源资产统计和审计改革。持续开展“绿满荆州”行动，新增城市绿地45公顷，森林覆盖率达到20.4%，荣获“全国绿化模范城市”称号。四湖流域生态修复工程稳步实施，洪湖、长湖拆除围网21万亩。石首市荣膺“中国江豚之乡”。

六是加快发展社会事业，增进了民生福祉。

扶贫攻坚卓有成效。扎实开展“三年扶贫攻坚行动”，产业、科技、医疗、教育、金融等扶贫工程有序推进。建立扶贫投入增长机制，统筹各类财政资金12亿元用于精准扶贫。落实结对帮扶、驻村帮扶全覆盖，建立健全贫困对象退出机制，全市建档立卡的121个贫困村、11.9万贫困人口如期脱贫。

基本民生得到保障。新增城镇就业8.34万人，城镇失业人员再就业3.04万人，就业困难人员实现就业1.37万人。发放创业担保贷款3.84亿元，扶持创业6153人。社会保险新增扩面10.7万人，城乡居民社会养老保险综合参保率99.6%。中心城区城市低保标准每人每月增加50元，农村低保标准每人每年增加800元。建成城乡居家养老服务中心959个。率先在全国开展未成年人社会保护工作试点。开工建设保障性住房4.4万套，基本建成2.9万套。中心城区放心早餐工程和菜市场改造顺利实施，公共厕所全面改造升级。十件民生实事全部完成。

社会事业协调发展。建成院士专家工作站50家，完成科技成果转化70项，专利授权量1850件。中心城区义务教育管理体制改革顺利完成，职业教育资源整合步伐加快。县级公立医院改革进展顺利，基层医疗卫生服务和重大疫病防控不断加强。食品药品监管基础进一步夯实。计划生育奖扶政策全面落实，出生二孩占比48.7%。现代公共文化服务体系加快构建，农村智能广播网基本建成，《好人开讲》走向全国，荆州花鼓戏《小城大爱》、现代歌剧《有爱才有家》深受好评。全民健身掀起热潮，全市乡镇体育设施覆盖率99.2%。“七五”普法全面启动。信访工作制度改革深入推进，一批信访积案妥善化解。开展“平安使命十大行动”，扎实推进立体治安防控体系建设，“一感两度两率”明显提升。国防动员、国防教育、国家安全、人民防空、民兵预备役工作得到加强。军民融合深度发展，“荆州舰”“洪湖舰”加入中国海军战斗序列，蝉联“全国双拥模范城”。

应急管理、防震减灾、对口支援、外事侨务、台湾事务、民族宗教等工作取得新成绩，机关事务管理、统计、物价、农垦、移民、烟草、供销、盐务、残联、慈善、气象、档案、社科、史志、保密和无线电等工作取得新进步。

各位代表！一年来，我们始终坚持以人民为中心，努力建设人民满意政府。办理本级人大代表建议134件、政协提案324件。全面推行依法行政，建立政府法律顾问和决策咨询制度。认真落实中央八项规定精神，扎实开展“两学一做”学习教育，从严从实整治“四风”等突出问题。效能监察和审计监督工作进一步加强。

值此换届之际，回首过去六年，荆州经济社会发展取得了一系列可喜变化。

——这六年，荆州战略定位大提升。随着沪蓉高铁建成通车、高速路网加快形成、江汉运河通水通航，荆州作为长江中游重要节点城市，积极融入长江经济带、长江中游城市群、洞庭湖生态经济区建设，区域战略地位更加凸显。省委、省政府大力支持荆州实施壮腰工程、加快荆州振兴，我市在全省“一主两副多极”格局中的份量正在加重。

——这六年，荆州综合实力大增强。我们成功实现了“六个千亿”“五个翻番”。地区生产总值、固定资产投资、社会消费品零售总额、招商引资到位资金、农产品加工业产值、金融机构各项贷款余额突破千亿大关。相比于2010年末，地区生产总值、固定资产投资、规模以上工业增加值、税收收入、地方公共财政预算收入实现翻番。

——这六年，荆州城乡面貌大变化。中心城区完成城建投资162亿元，先后获批国家园林城市、国家生态文明建设先行示范区、国家智慧城市试点。多年的大投入、大建设，提升了荆州的对外形象和城市品位。我们生活的这片乡土，正在以前所未有的加速度，变得更加靓丽、更加宜居、更加美好。

——这六年，荆州民生福祉大改善。累计民生支出1361亿元，占地方公共财政预算支出的80.6%。居民收入稳定增长，连续六年跑赢GDP。荣获全国创业先进城市，城镇新增就业人员总数42万人，城镇登记失业率持续控制在4.5%以内。社会保障和救助体系基本实现全覆盖。

——这六年，荆州干群士气大提振。我们争先进位，打破了在全省发展靠后的沉闷局面。我们齐心协力，圆满承办湖北省第十四届运动会，展示了荆州形象。我们全程接力，积极参与“东方之星”号客轮救援处置，谱写了人间大义大爱。我们众志成城，奋力抗击特大洪涝灾害，实现了抗洪保安全、保丰收、保发展。我们全城动员，取得了创建国家卫生城市的阶段性胜利。实践证明，荆州的干部群众每临大事有定力，每遇难事敢担当，越是在困难和压力面前，越能团结一致、合力攻坚。

各位代表！过去六年取得的成绩，是省委、省政府和市委正确领导、科学决策的结果，是全市广大干部群众上下一心、开拓奋进的结果。在此，我代表市人民政府，向全市人民，向老干部、老领导，向驻荆部队、武警官兵、政法干警和各级驻荆单位，向所有关心支持参与荆州建设发展的各界人士，表示衷心的感谢，并致以崇高的敬意！

各位代表！在肯定成绩的同时，我们也清醒地认识到，我市经济社会发展中仍然存在一些困难和问题：经济总量不大，人均水平不高，产业集群发展滞后，服务业层次低，产业结构不优，创新能力不足，综合竞争力不强；发展方式粗放，生态环境约束趋紧，生产要素成本上升；城市功能不完善、管理不精细，城乡统筹水平不高，全面建成小康社会还存在短板；协调各方利益、维护社会稳定压力大，安全生产、污染治理、交通拥堵、食品安全形势严峻；洪监高速等部分重点工程进展不快。同时，政府职能转变有待深化，政府工作人员干事创业的担当精神、为民解难的服务意识和雷厉风行的工作作风有待加强。对这些问题，我们要高度重视，采取积极有效措施，切实加以解决。

二、未来五年发展目标

各位代表！未来五年，是全面建成小康社会的决胜阶段，是实现荆州振兴的关键时期。我们既处在发展环境、发展条件的深刻变化期，更处在深化改革与加快转型的机遇期。从宏观环境看，经济新常态下，我国经济发展总体平稳、稳中向好，为我们深化改革扩大开放、推进经济结构调整和产业转型升级提供了良好条件。从发展机遇看，随着“一带一路”、长江经济带、中部崛起、长江中游城市群、洞庭湖生态经济区等国家战略的深入实施，荆州作为区域重要节点城市大有可为。省委、省政府支持荆州建设区域中心城市，为我们在全国全省发挥更大作用提供了战略空间。从自身基础看，历经五年壮腰实践，我们打下了坚实基础，培育了综合优势，营造了政通人和、齐心干事的良好局面，必将强力推动荆州加快发展。

未来五年，我们要高举中国特色社会主义伟大旗帜，以马克思列宁主义、毛泽东思想、邓小平理论、“三个代表”重要思想、科学发展观为指导，全面贯彻党的十八大和十八届三中、四中、五中、六中全会精神，深入学习贯彻习近平总书记系列重要讲话精神和治国理政新理念新思想新战略，认真贯彻落实市第五次党代会精神，紧紧围绕统筹推进“五位一体”总体布局和协调推进“四个全面”战略布局，践行新发展理念，积极适应和引领经济发展新常态，以供给侧结构性改革为主线，深入实施壮腰工程，把荆州建设成为湖北经济新的增长极、江汉平原现代化中心城市和长江中游重要中心城市，奋力实现荆州“十年大振兴”的宏伟蓝图。

未来五年，我们的发展目标是：打造“一极两中心”，实现“十年大振兴”。

——打造“新的增长极”：经济总量跃上3000亿元台阶，主要经济指标年均增速保持在全省第一梯队，打造湖北经济新的增长极。

——建设“两个中心”：统筹区域协调发展，努力将荆州建设成为江汉平原现代化中心城市、长江中游重要中心城市。

未来五年，我们的工作思路是：

——共抓大保护。把修复长江生态环境摆在压倒性位置，“共抓大保护，不搞大开发”。对接洞庭湖生态经济区建设，实施绿色发展战略，探索低碳、循环、可持续发展路径，以对人民群众、对子孙后代高度负责的态度，以最严密的制度、最严格的管理、最严厉的执法，全面推进生态文明建设。大力实施“蓝天、碧水、净土、绿地”工程，进一步筑牢生态屏障，呵护江河湖泊，守护黑土地。

——构筑大交通。把“大交通”作为厚植发展优势的加速器。推进沙公高速南延线、江北高速东延线、二广高速改线等工程，建设依托沙公高速、沪蓉高速和襄荆高速的中心城区内环，建设依托荆州长江大桥、荆州长江公铁两用桥的高速公路跨江外环，形成“二环三横六纵”市域快速通道。加快国省干道改造升级，形成布局合理、互联互通的公路骨干网；进一步打造“一港十区”沿江港口群；建设荆州机场和2-3个通用航空机场；推进“两横两纵”铁路网建设，构筑衔接顺畅、功能完善的综合运输体系，打造长江中游具有铁水公空联运功能的综合交通枢纽城市和国家区域级流通节点城市。

——培育大产业。把“大产业”作为引领发展的“牛鼻子”。落实扶持实体经济发展政策，实施千亿产业、千亿园区工程，进一步壮大食品加工、装备制造、医药化工3个千亿产业和电子信息、轻工建材、纺织服装3个五百亿产业，促进产业集群发展。实行一个产业一个园区、一个产业一个规划，重点对接带动力强的大企业、大集团和上下游关联企业，按照规划定位推进产业招商、精准招商。把文化旅游作为荆州换道超越的引爆点，突破性发展文化旅游业。创建国家级华中农高区，实施创新农业，推动农业大市向农业强市转变。

——建设大城市。把“大城市”作为推动发展的主载体，推进以人为核心的城镇化。科学编制中心城区、县市城区、中心镇、特色镇等功能明确的城镇体系规划，推进城乡协调发展。统筹“一江两岸”，促进江陵、公安与中心城区融合联动发展。支持松滋、石首、监利、洪湖竞相发展，促进人口集聚、功能提升和规模合理扩张，建设中等城市。以荆州开发区、纪南文旅区、华中农高区和荆北、沙北片区为引领，以“五城同创”倒逼城市规划、建设、管理升级，增强城市意识，塑造城市特色，努力建设美丽家园、平安城市、幸福荆州。

三、2017年重点工作

2017年，是实施“十三五”规划的重要一年，是供给侧结构性改革的深化之年。今年经济社会发展的主要预期目标是：地区生产总值增长8.5%，规模以上工业增加值增长9%，固定资产投资增长16%，社会消费品零售总额增长12%，外贸出口增长10%，地方公共财政预算收入增长8%，城乡居民人均可支配收入均增长8.5%以上，城镇登记失业率控制在4.5%以内，完成省下达的节能减排任务。

（一）着力优化经济结构。

以扩增量、提质量为根本，推动工业转型升级。坚持把改造传统产业与引进新兴产业放到同等重要的位置，在解决我市工业“小、散、低”问题上内外并举、双管齐下。改造提升传统产业。制定六大主导产业规划，完善“一区多园”管理体制机制，引导产业、资金、技术向全域同类园区集聚。鼓励、引导企业加大技改投入，推进“两化融合”，促进农产品加工、医药化工、纺织服装、轻工建材等传统产业提质增效。以荆州开发区为主战场，以公安、洪湖、石首汽车产业集群为支撑，大力发展汽车零部件产业集群，谋划汽车整车制造。支持江陵县发展新型煤电煤化产业集群。发展壮大新兴工业。全面对接“中国制造2025”，重点支持新能源汽车、新一代光电子信息、智能制造、生物医药、海洋工程装备、新材料、节能环保等战略性新兴产业发展壮大，不断提升高新技术产业比重，新增高新技术企业30家以上。建设军民融合产业园，推进军民融合产业发展。弘扬“工匠精神”，实施“质量强市”战略，开展消费品工业增品种、提品质、创品牌“三品”专项行动，以优质产品、名牌建设引导产业结构调整和资源配置。激活市场主体。发挥担保平台、“助保贷”“新农贷”、续贷调度资金、小额贷款保证保险补偿金作用，重点支持科技小巨人、中小微企业“小进规”“规改股”“股上市”，确保净增规模以上企业100家。继续推进福娃集团“211工程”、中兴能源“222工程”、白云边集团“2211工程”，扶持沙隆达、凯乐、恒隆、能特、四机、拍马等企业冲刺百亿元产值，支持荆州美的打造全球第一冰箱生产基地。

以华中农高区为引领，大力发展现代农业。更加重视农业基础地位，深入推进农业供给侧结构性改革，加快转变农业发展方式，打造市场农业、规模农业、科技农业、生态农业，促进一二三产业融合发展。加快创建百亿斤粮食大市。实施江汉平原国家级土地整治工程，新建高标准基本农田51万亩。推广“福娃模式”，引导土地加快流转，实施稻渔综合种养、集中育秧、机械插秧和超级稻种植技术，新增粮食产量3亿斤。实施绿色农产品生产示范工程，推进畜禽标准化、专业化、规模化生产，发展生态渔业、品牌渔业、效益渔业，巩固荆州国家淡水渔业第一市地位。加快发展农产品加工业。支持华中农高区创建国家级农高区，建立20家以上院士专家工作站、省级以上研发中心、实验室，引进科研院所建设科技成果转化基地，建设江汉平原农业科技创新中心。实施“农产品加工业倍增计划”，支持“荆楚味道”区域农业公共品牌扩大影响，发展壮大一批市场竞争力强的龙头企业，力争新增农产品加工业产值200亿元。加快培育农业新型经营主体。鼓励专业大户成立家庭农场，引导高校毕业生、个体工商户、农村经纪人等投身现代农业开发，力争新注册家庭农场500个。发展农业职业教育，培育新型职业农民。落实财政奖补资金，实施“三建三联”，鼓励农民专业合作社组建联合社，打造一批大社强社。完善农村土地承包经营权确权登记颁证工作，实现农村土地承包管理信息化、常态化。落实承包土地“三权分置”办法，推动农村产权制度改革。

以文化旅游、金融工程为重点，加快繁荣现代服务业。继续推动服务业发展提速、比重提升、结构优化。壮大文化旅游业。争创中国书法城市、中国诗词之市、中国楹联文化城市，力争每年创作一部与荆州有关的戏曲，拍一部与荆州有关的电影、电视剧。把荆州作为旅游目的地来建设，把文化旅游业作为战略性支柱产业来培育，把全域旅游作为供给侧结构性改革的重头戏。支持纪南文旅区加快建成文化旅游的“中国品牌、湖北代表”。抓好荆州古城五环5A创建，升级松滋洈水、石首天鹅洲、荆江南北闸、洪湖岸边是家乡等旅游板块，推进华强方特园、海洋世界、文博园、古城保护与修复等项目建设。发展乡村旅游，积极开发工业、农业、健康养生等特色旅游。加大宣传推介力度，打造荆州旅游品牌。壮大商贸物流业。推进大型商贸流通项目建设，加快发展仓储、货代、联运、快递。支持国家级荆州淡水产品交易中心建设。加快传统商贸服务业提档升级，发展区域零售企业集团，加强农村市场体系建设。发展电子商务、云计算服务、物联网服务、第三方物流、融资租赁、信息咨询、节能环保、检验检测、服务外包等新兴产业和医疗、卫生、体育、养老、法律咨询等生活性服务业。壮大现代金融业。开展金融招商，对接引进各类金融机构。发挥产业引导基金作用，推动更多基金项目和产业项目落地。抓好企业债发行工作。分类实施县域金融工程“普及版”和“升级版”。鼓励有条件的企业在境内外上市、在“新三板”挂牌，新增“新三板”挂牌5家、“四板”挂牌300家。积极防范和化解各类金融风险。

（二）着力培植竞争优势。

加快重大基础设施建设。精准对接国家、省重点投资领域和专项建设基金投资方向，谋划储备一批重大项目，争取更多项目纳入国家和省级规划。按照市直4-5个、县市2-3个的标准，从PPP项目库中遴选一批项目纳入年度推进计划。加快建设沙公高速、洪监高速、石首长江大桥、洪湖嘉鱼长江大桥。开工建设江北高速东延线、洪湖赤壁长江大桥、沪渝高速中心城区服务区和小北门互通、沙市互通、八岭互通改造工程，谋划江北高速西延。推进荆州民用机场、纪南通用机场、城北快速、松滋车阳河码头二期、江汉平原铁路建设。加快洪湖东分块蓄滞洪区、后三峡长江河道整治工程建设，整险加固洪湖、长湖围堤，改造大中型涵闸、泵站。

全面深化改革。落实“1+4+8+N”专项改革行动方案，推进简政放权、减负降费，营造公平竞争市场环境。实施“互联网+政务服务”，加快公共服务信息平台建设，促进部门公共服务互联互通、信息共享。深化财政改革，编制中期财政规划。建立乡镇政府权力清单和责任清单制度。整合检验检测机构。推进价格机制改革。实现国有经营性资本监管全覆盖。抓好投融资体制改革，做实市城投、农高投、海投公司等政府投融资平台，发展创业投资、天使投资等创新投资机构，引导和鼓励社会资本进入公共服务领域。稳步推进事业单位公车改革。

积极推动“大开放”。扩大双向开放，以大开放促进大发展。树立世界眼光，坚持出口和进口并重，“引进来”和“走出去”并重，坚定不移引进外资和外来技术、外籍人才，积极建立友好城市，厚植开放。瞄准世界500强、央企、国企、大型民企，开展园区招商、产业招商、存量招商、基金招商，力争实际到位资金增长10%。引进大型企业集团总部落户荆州，加快发展总部经济。支持恒隆、沙隆达等优势企业开拓境外市场。鼓励、支持、引导民营经济加快发展。积极发展跨境电商。抢抓武汉自贸区建设机遇，加快荆州水运口岸对外开放，积极申报综合保税区。加快融入“一带一路”、长江经济带，深化与相关城市在金融、港航、产业、社会建设等方面的合作。启动建设临空临港经济区。

深入推进“大众创业、万众创新”。围绕产业链，实施“校（院）企联姻”工程，共建重点实验室、校企研发中心、产业研究院等创新平台。支持恒润科技孵化器、速乐购争创国家级众创空间。推进开发区建设大型公共创新创业园，支持引导在荆高校建设校园科技孵化器。开展劳动技能竞赛，激发广大劳动者创业创新热情。实施大学生创业引领计划、外出务工人员等群体返乡创业计划，加强创业广场项目、创业孵化基地和创业公共服务体系建设。支持市创业学校建设以非遗传承为特色的高职院校。

（三）着力提升城乡发展水平。

提升中心城区首位度。适时修改城市总规，调整完善土地利用总规，加快实现“多规合一”。保护历史遗产，留住荆州“乡愁”。坚持以“五城同创”为抓手，倒逼中心城区布局更合理、功能更完善。一举创成国家卫生城市，继续开展国家生态园林城市、国家森林城市、省级环保模范城市、省级文明城市创建。加快建设荆州大道、凤凰大道、楚都大道、海子湖大道、318国道改扩建工程。推进“滨长江、环古城、沿长湖、伴运河”城市景观带建设，启动园博园建设，加快建设植物园、城南公园、白水滩湿地公园、环荆州古城国家湿地公园。以更大力度开展棚户区改造。加快城市地下空间和综合管廊建设，打造海绵城市。建成“荆楚云”，完善智慧交通、城管、社区、健康、旅游、教育等项目设计方案，加快建设智慧城市。推进城市执法体制改革。

统筹县域城镇发展。依据区位、人口、资源禀赋等不同条件，合理实施规划布局，加快推动产城融合，形成城镇体系结构合理、区域发展协调、城乡一体化发展的新型城镇带。加快户籍制度改革，促进城镇基本公共服务向常住人口全覆盖。依法维护进城农民土地承包权、宅基地使用权和集体收益分配权，调动广大农业转移人口进城落户的积极性。有序供应建设用地，促进房地产市场平稳健康发展。

建设美丽乡村。完成省级以上特色镇、各类试点示范镇镇域规划、控制性详细规划编制工作。推进松滋洈水、卸甲坪和监利新沟、程集首批美丽乡村建设试点。推进农村绿化净化美化，延伸农村绿色版图，建设生态乡镇30个、生态村600个。实施乡镇生活垃圾无害化处理，加强乡镇污水处理厂建设管理与管网配套。新建和改建农村公路800千米。改造升级农村电网，完善农村医疗、文化、体育、邮政、通信、环保等基础设施。

（四）着力改善生态环境。

加强生态建设。坚持生态优先，走可持续发展道路。加快实施四湖流域综合治理和洞庭湖流域水污染防治工程，推进江河湖水系连通工程，疏通治理大中型湖泊、水库、河渠，加快退田、退垸、退渔还湖。各县（市、区）至少恢复2个以上湖泊，并申报创建国家级、省级湿地公园。扎实开展“绿满荆州”行动，加快松滋、江陵、公安、石首、监利、洪湖省级森林城市创建，建设树木园、森林博物馆、长江生态防护林、城市小森林，推进农田林网、通道、水源地绿化。

深化环境整治。继续开展“三禁两治”，加强重点行业工艺尾气和挥发性有机物污染治理，提标改造重点行业脱硫和除尘设施，完成省下达的年度空气质量考核指标，让蓝天白云常驻荆州。坚持“三污同治”，切实加强工业污染源环境管理，确保重点涉水企业污水治理设施正常运行，稳定达标排放。开展重点流域、区域水环境综合整治，完成城区雨污分流管网建设，提高城市污水收集处理率。实施农业投入品减量化。

强化节能降耗。从严控制用能总量，坚决淘汰落后产能，确保完成节能降耗各项指标。全面实施清洁生产，推进资源综合利用和循环经济发展。切实抓好工业、建筑、交通等重点领域和公共机构节能监管。加强节能宣传，提高全社会节能意识。

（五）着力增进民生福祉。

狠抓精准脱贫。找准“穷根”，对症下药。全力开展扶贫“九大工程”。按照“一村一策、一户一案”要求，整合财政资金，加大金融扶贫力度，引导贫困村、贫困户与农业龙头企业、农民专业合作社、家庭农场和种养大户建立利益联结机制。完成113个贫困村、82477名贫困人口的年度减贫任务。推进共享，促进全面小康。

加强社会保障。新增企业职工养老参保3.1万人，城镇职工医疗保险和城乡居民基本医疗保险参保人数达到应参保人数的95%以上。落实城乡低保标准合理增长机制。新增住房公积金归集额20亿元，发放公积金个人贷款12亿元。通过租赁补贴和实物配租等方式，确保全市低收入住房困难家庭应保尽保。

发展社会事业。实施全民科学素质行动计划纲要。加快教育信息化、学校标准化建设步伐，提升教育均衡发展水平。规范学前教育管理。优化调整中心城区学校布局，完成荆州中学、沙市中学、荆州体校搬迁。坚持与长江大学融合发展，支持其植根荆州，建设全国一流大学、一流学科。实施职业教育资源整合工程，加快荆州职业技术学院新校区建设，推进全国现代学徒制改革试点，培育“荆州工匠”。继续打造大型群众体育品牌赛事，促进群众体育运动蓬勃发展。完善突发性公共卫生事件应急机制，建成疾病预防控制体系。加快市、县级公立医院改革步伐，推进鄂中南区域医疗中心、市一医门诊综合大楼等项目建设，加强妇幼保健医院、中医院建设。强化人口与计划生育工作目标管理，落实二孩生育政策。加强血吸虫病综合防治。保障妇女儿童合法权益。开放市图书馆新馆，抓紧荆州剧院、城市规划展览馆、档案馆新馆、群艺馆新馆规划建设，启动市委党校和科技馆搬迁规划，加快县级“两馆”建设。推进基层综合性文化服务中心建设。推进广播电视整合。建立健全非遗保护利用机制，加强文物古迹的保护和申遗工作。实施食品放心工程，构建食品安全标准体系。维护社会稳定，突出风险防控和社会稳控，深入推进“平安荆州”“法治荆州”建设，加大“两抢一盗”、非法集资、网络电信诈骗等案件防范打击力度，构建立体化社会治安防控体系，争获全国社会治安综合治理“长安杯”。坚持依法信访，做好群众来信来访和人民调解工作，妥善处理和化解人民内部矛盾。加强安全生产专项治理，加大事故隐患整改力度。加强国防、人防和民兵预备役建设。发展老年人、残疾人事业，进一步做好外事侨务、台湾事务、民族宗教、统计、物价、农垦、移民、烟草、供销、盐务、慈善、气象、档案、社科、史志、保密和无线电等各项工作。

办好民生实事。一是新增城镇就业6.8万人，城镇失业人员再就业2万人，就业困难人员再就业1万人，就业创业培训1万人，扶持创业5000人，发放小额担保贷款2亿元；免费培训新型职业农民4600人。二是实施棚户区改造4.67万套，基本建成2.33万套。三是在中心城区试点建设30个机关与市民共享的图书阅览室，为112个社区配送图书；在120所农村中小学实施“希望书屋”项目，推进“全民阅读”。四是淘汰中心城区规划区80台高污染燃料锅炉。五是在中心城区建设30家放心早餐示范店和放心早餐店。六是免费为100名贫困下肢残疾人安装假肢，为300名贫困白内障患者实施复明手术；免费为500名贫困残疾儿童实施康复救助；为5.7万名贫困残疾人、4.7万名重度残疾人发放生活和护理补贴。七是易地扶贫搬迁贫困渔民上岸1605户5973人。八是为经济困难群众和农民工办理法律援助案件3000件，办理法律援助事项3万件。九是建设100个省级绿色示范乡村。十是新建改造配电台区1600个，新建改造10千伏及以下线路6700千米。十一是在中心城区新建停车场7个，新增停车泊位2800个。

（六）着力加强政府自身建设。

推进依法行政。牢固树立法治思维，恪守合法行政、程序正当、权责统一等依法行政基本要求，深入开展“七五”普法工作，带头信仰法律、尊崇法律。坚持党的领导，自觉接受人大法律监督、工作监督及政协民主监督，主动接受司法监督、社会监督和舆论监督，广泛听取各民主党派、工商联和无党派人士的意见和建议，认真办理人大代表建议和政协提案。完善法律顾问制度，健全政府依法决策机制，加强合法性审查，发挥市政府决策咨询委员会作用，开展重大行政决策风险评估，做到依法决策、科学决策。健全政府信息公开制度，依法公开重点领域政府信息，让行政权力在阳光下运行。

坚持务实重行。切实增强政治意识、大局意识、核心意识、看齐意识，少说多干、埋头苦干、科学巧干、合力大干，察实情、说实话、办实事、求实效。不提新战略、不喊新口号、不提超越阶段的目标、不做不切实际的事情，满怀信心、不忘初心，保持定力、精准发力，一步一个脚印把事业推向前进。以“功成不必在我”的境界和历史耐心，多做打基础、管长远、利发展、惠民生的事情。严格绩效管理，深化治庸问责，政府及政府部门只有落实的义务、没有推诿的权力。凡是市委、市政府议定的决策事项，都要工程化、项目化、具体化，明确“时间表”，制定“施工图”，落实责任人，坚决杜绝有令不行、有禁不止。健全正向激励和容错纠错机制，构建“亲”“清”政商关系。

严守廉洁底线。严格落实党风廉政建设主体责任和“一岗双责”，深入推进政府系统廉政建设和反腐败工作。加强行政监察和审计监督，坚持有腐必反、有贪必肃、违规必究。持续开展廉洁警示教育活动，切实加强工程建设、土地出让、政府采购、产权交易等重点领域监管，严肃查处各类违法违纪行为和发生在群众身边的腐败问题。厉行节约、反对浪费，严控“三公”经费支出，把更多财力用在“刀刃”上、用在民生事业上。

各位代表！政府的权力来自人民，必须对人民负责，为人民谋福祉。我们将始终坚持以人民为中心，及时回应群众关切，努力为人民服务、为发展服务，扎实办好荆州的事情，建设人民满意的政府。

各位代表！肩负全面建成小康社会的历史重任，我们又踏上了新的征程。让我们紧密团结在以习近平同志为核心的党中央周围，在省委、省政府和市委的领导下，凝心聚力，奋发有为，不断开创各项事业发展新局面，为建设“一极两中心”，实现荆州“十年大振兴”而努力奋斗！
